# Impaired Lung Function and Quality of Life Outcomes in Patients with Tuberculosis: A Cross-Sectional Study

**DOI:** 10.3390/tropicalmed10090247

**Published:** 2025-08-29

**Authors:** Varshini Jagadeesh, Prashanth Chikkahonnaiah, Muskan Dubey, Shashidhar H. Byrappa, Hari Balaji Sridhar, Raghavendra G. Amachawadi, Ravindra P. Veeranna

**Affiliations:** 1 Department of Pulmonary Medicine, Mysore Medical College and Research Institute, Mysuru 570001, Karnataka, India; varshinijagadeesh@gmail.com (V.J.); prshnthcr@gmail.com (P.C.); 2 Xavier University School of Medicine, Xavier University School of Veterinary Medicine, Santa Helenastraat #23, Oranjestad, Aruba; muskan.dubey@students.xusom.com; 3 Department of Pathology, Mysore Medical College and Research Institute, Mysuru 570001, Karnataka, India; shbmysore@gmail.com; 4 Department of Clinical Sciences, College of Veterinary Medicine, Kansas State University, Manhattan, KS 66506, USA; haribala30@vet.k-state.edu

**Keywords:** tuberculosis, quality of life, drug-resistant tuberculosis

## Abstract

Tuberculosis (TB) continues to be the world’s deadliest infectious disease, with an estimated 10.8 million new cases reported in 2023, of which India alone accounted for 28% of the global burden. This study aims to evaluate the impact of tuberculosis on pulmonary function and exercise tolerance, and to examine how these impairments affect health-related quality of life (HRQoL). In a cross-sectional design, 96 bacteriologically confirmed TB patients and 96 age- and sex-matched community controls underwent spirometry, six-minute-walk test (6 MWT), and HRQoL evaluation. DR-TB was detected in 27 patients (28.1%): Isoniazid monoresistance 59.3%, rifampicin monoresistance 11.1%, and XDR-TB 29.6%. Dyspnoea (70.8%) and cough (37.5%) were the most commonly reported symptoms among TB patients. Mean values of FEV_1_, FVC, and FEV_1_/FVC were significantly lower in TB patients compared to controls (62.8%, 65.97%, and 70.08% vs. 82.55%, 80.09%, and 78.08%, respectively; *p* < 0.001). Recurrent or DR-TB was associated with reduced spirometric indices and 6 MWT distances (241 m vs. 358 m in drug-sensitive TB). St. George’s respiratory questionnaire (SGRQ) scores indicated significantly poorer health-related quality of life (HRQoL) in patients compared to controls across all domains—symptoms (23.7 vs. 10.7), activity (33.3 vs. 14.2), and impact (20.6 vs. 9.4; *p* < 0.05). SGRQ scores were inversely correlated with lung function parameters (r = −0.42 to −0.56). These findings underscore the persistent health burden TB poses post-therapy, highlighting the need for routine post-TB functional screening and robust DR-TB control to achieve End-TB goals.

## 1. Introduction

Tuberculosis (TB) remains the leading cause of death from a single infectious agent worldwide, surpassing HIV/AIDS. Tuberculosis (TB) poses a significant global public health challenge, remaining a leading cause of morbidity and mortality, with approximately 4000 deaths occurring each day. Despite progress in detection and treatment, around 10.8 million individuals have contracted TB, and roughly 1.2 million people have passed away from TB worldwide in 2023 [1]. Approximately one-quarter of the world’s population has been infected with Mycobacterium tuberculosis at least once during their lifetime. TB patients can display a wide variety of symptoms, ranging from subclinical infection to those affecting one organ or multiple organ systems. The most frequently reported symptoms are cough and fever [2]. Patients are recognized to experience distress from disease symptoms, with resulting deterioration in health-related quality of life (HRQoL) [3].

The END TB strategy by the World Health Organization (WHO) strives to decrease TB deaths by 95 percent and lower new TB cases by 90 percent by the year 2035 [4]. Achieving global targets for reducing the tuberculosis (TB) burden requires substantial improvements in TB diagnostic, preventive, and treatment services [5]. Geographically, among the six TB high-burden countries in the Southeast Asia region, India represents 28 percent of the global TB burden [1]. According to the National TB Prevalence Survey conducted in 2021, the crude prevalence of tuberculosis among individuals aged over 15 years was 31.3% (95% confidence interval [CI]: 30.8–31.9). Of these individuals, an estimated 5–10% are expected to progress to clinically active TB disease [6]. Due to delays in diagnosis, a single case of active tuberculosis can transmit the infection to multiple individuals before treatment initiation, thereby sustaining a continuous reservoir of TB cases [7]. The treatment of TB is often underestimated in its role in preventing the disease, yet it remains a crucial component of the National Strategic Plan 2017-25 aimed at ending TB in India by 2025, which is five years earlier than the sustainable development goals [8].

Despite recent medical and health advancements, TB has successfully avoided effective targeting by medications by evolving resistance towards antibiotics. Drug-resistant tuberculosis encompasses several forms, including multidrug-resistant (MDR), pre-extensively drug-resistant (Pre-XDR), and extensively drug-resistant (XDR) TB [9]. In 2021, an estimated 450,000 new cases of multidrug-resistant tuberculosis (MDR-TB) were reported, representing a 3.1% increase from 437,000 cases in 2020 [10].

In various countries, MDR strains represent as much as 20% of new TB instances and more than 50% of individuals with a background of prior TB therapy. In 2006, the term XDR-TB was created to refer to MDR-TB strains that are resistant to fluoroquinolones and second-line injectable medications. Interestingly, about 9.6% of MDR-TB cases worldwide are classified as XDR-TB [11]. Another crucial element of MDR-TB treatment is tackling socioeconomic factors affecting health. Socioeconomic issues, such as hunger, lack of housing, and unemployment, are prevalent among MDR-TB patients. Tackling socioeconomic obstacles to adherence is essential for effective MDR-TB treatment in a community.

Pulmonary TB (PTB) may result in chronic airflow blockages based on the extent of anatomical changes, and restricted ventilation due to fibrotic scarring linked to diminished total lung capacity. Severe lung injury, linked to disability and reduced quality of life, has been connected to a delayed TB diagnosis, the number of previous treatments, smoking, malnutrition, and a high bacillary load at the start of anti-TB therapy (ATT) [12]. The functional alterations observed post-treatment from PTB present as restrictive lung disease (RLD), obstructive lung disease (OLD), or a combination of both, known as mixed obstructive–restrictive lung disease (MORLD), irrespective of smoking exposure history [13].

Quality of life (QoL) is a multifaceted concept encompassing physical, social, mental, economic, and other dimensions. It reflects an individual’s overall satisfaction and is often assessed based on the patient’s own evaluation of their health status, functional ability, and general well-being [14]. Since QoL highlights an “individual’s view of their status in life based on the value systems and culture they inhabit, along with their expectations, objectives, benchmarks, and worries,” it poses challenges in evaluation. Consequently, instead of evaluation by healthcare professionals, QoL is thought to represent the preferences and values of the patient. Since HRQoL reflects patients’ awareness of their mental and physical well-being, it is crucial for understanding and quantifying the true effect of the illness [15]. Indeed, several studies have ascertained the association between pulmonary functioning and QoL in both acute and chronic disorders. In a study to assess the effects of aging on lung function and QoL, it was shown that poor pulmonary function was associated with poor general health and reduced physical and cognitive performance [16]. It was found that patients with severe obstructive form of cystic fibrosis had poor QoL as compared to patients suffering from a milder obstructive form of the disease [17]. Similar results were observed in another study where the association between pulmonary function, QoL, and exercise tolerance was studied [18].

This is not surprising as most of the pulmonary disorders are associated with poor lung function, with subsequent impact on QoL. It was found that patients suffering from exacerbations in chronic obstructive pulmonary disease (COPD) were found to have poor total sleep time, reduced sleep efficiency, and higher levels of fatigue, owing to the severe pulmonary symptoms during episodes of exacerbation, impacting the overall QoL in these patients [19,20]. Patients suffering from COPD also have a high frequency of hospitalization, which could also lead to functional and physical impairment in patients [21]. Due to severe symptomatic restraint on patients with COPD exacerbations, patients have poor muscle strength and dyspnea, which leads to a high rate of falls following hospitalization [22]. Moreover, patients with COPD are at increased risk of depression and anxiety, which are often exacerbated by frequent hospitalizations and the severity of disease symptoms [23,24].

Various studies have made efforts to provide evidence behind the association between impaired pulmonary function and consequent QoL impacts in TB. One of the important hallmarks of TB is severe somatic symptoms characterized by weight loss and weakness [25]. Another major impact TB has on QoL is the social stigma associated with it, on the level of both the family and community [26,27]. This is due to the fact that TB, being a highly transmissible disease and often associated with human immunodeficiency virus (HIV) infection, is often stigmatized by community members [28]. People diagnosed with TB have often reported social isolation and loss of friends, contributing to depression and reduced QoL [29]. Diagnosis of TB is often associated with fear of social boycott and seclusion, with depressive symptoms like anorexia, loss of body weight, and general tiredness [30]. Frequent hospitalizations and cost of treatment associated with TB led to a severe financial burden on patients’ families, with subsequent impact on health and social welfare [27]. Effect of ATT on patients’ QoL was found to be generally varied based on duration of therapy, type of ATT, and cost of treatment. Several studies have noted that patients undergoing ATT had significant improvement in physical and mental well-being, owing to general improvement in pulmonary function [31,32,33,34]. Thus, it is evident that TB infection negatively impacts every aspect of quality of life, spanning physical, economic, and social areas, along with psychological suffering due to the stigma and discrimination linked to the condition [31,35].

Health-related quality of life (HRQoL) has become a critical outcome measure for patients with respiratory diseases, as evidenced by the development of numerous disease-specific HRQoL questionnaires in recent years. Among these, the St. George’s respiratory questionnaire (SGRQ) is one of the most widely used instruments for assessing HRQoL in respiratory patients and has been adapted into multiple languages [36]. The St. George’s respiratory questionnaire (SGRQ) has been extensively utilized in both descriptive studies and therapeutic evaluations, including assessments of bronchodilator medications, oxygen therapy, psychotherapy, and pulmonary rehabilitation. However, its use remains largely confined to research settings. Several limitations hinder the routine application of HRQoL assessments in clinical practice, with the interpretation of HRQoL outcomes identified as a major barrier to their broader clinical implementation [36].

The St. George’s respiratory questionnaire (SGRQ) is a tool tailored to evaluate individuals with respiratory and immune system conditions, particularly asthma, lung diseases, and chronic obstructive pulmonary disease. SGRQ includes 50 questions spread across three areas of interest (symptoms, activity, and effects on daily life). Scores range from 0 to 100, with elevated scores reflecting poorer HRQOL [37,38]. The SGRQ is a standardized self-administered respiratory disease-specific questionnaire divided into three subscales: symptoms (8 items), activity (16 items), and impacts (26 items) [36].

Thus, our main objectives in the study were to assess the prevalence of TB among patients and to estimate the extent of pulmonary damage and function loss among TB patients and its effect on the QoL of patients.

## 2. Materials and Methods

### 2.1. Study Setting

The study was conducted at the Department of Respiratory Medicine, Princess Krishnajammanni Tuberculosis and Chest Diseases (PKTB and CD) hospital, Mysore Medical College and Research Institute (MMC&RI), Mysore.

### 2.2. Study Design

This was an institution-based cross-sectional study of subjects, conducted from December 2019 to May 2021.

### 2.3. Study Population

The study included consenting, adult (≥18 years old) patients who had a history of suffering from TB and had successfully completed a course of TB treatment as per national guidelines. TB patients who did not consent and those with an irregular treatment course were excluded from the study. All persons with pre-existing illness, extra-pulmonary TB, and those with HIV-TB coinfection were also excluded. Similarly, those patients with spirometry values who did not fulfill the acceptability and reproducibility criteria of spirometry as per American Thoracic Society (ATS) guidelines were also excluded from the study. Healthy volunteers were selected randomly as controls in the study.

### 2.4. Ethics

The study was conducted in accordance with the Declaration of Helsinki and received approval from the Institutional Ethics Committee of Mysore Medical College and Research Institute (MMC&RI), Mysore, Karnataka, India (Reference number: ECR/134/Inst/KA/2013/RR-16).

### 2.5. Study Tools and Protocol

Clinical history and physical examination of the subjects were thoroughly performed. A St. George’s respiratory questionnaire was undertaken by the subjects to measure the components of HRQoL. The components in the questionnaire include symptoms (8 items), activity (16 items), and impacts (26 items). Based on the responses to the questionnaire, a total and three different components’ scores were obtained. Scores from the questionnaire usually range between 0 (apparently healthy) and 100 (relatively worst health).

Spirometry was conducted on both the subjects and controls using the CMSP-01 spirometer, following the American Thoracic Society (ATS) guidelines. Pulmonary function test (PFT) parameters, including forced expiratory volume in one second (FEV_1_), forced vital capacity (FVC), and the FEV_1_/FVC ratio, were recorded. Each PFT value was expressed as a percentage of predicted population values based on age, sex, height, weight, and body surface area. A 6 min walk test (6 MWT) was conducted according to ATS recommendations to assess the functional capacity of the study subjects.

### 2.6. Statistical Analysis

The sample size required for the study was calculated using the estimation technique for proportion by

$$N=\frac{Z^{2}pq}{d^{2}}$$

*Z* = standard normal variate at 5% level of significance;

*p* = prevalence outcome (48%) [38]

*q* = 1 − *p*; *d* (absolute allowable error = 10%)

$$N=\frac{3.84\times 0.48\times 0.52}{(0.1{)}^{2}}=96$$

The estimated sample size for this study was found to be 96 in number.

Data from 96 subjects and 96 controls were compiled and organized using Microsoft Excel. Statistical analyses were performed using IBM SPSS Statistics software, version 23.0, IBM Corp., Armonk, NY, USA. Univariate analyses included the chi-square test for categorical variables, the independent samples *t*-test for comparison of means between groups, and Pearson’s correlation coefficient to assess relationships between continuous variables. A significance level of *p* < 0.05 was considered statistically significant.

## 3. Results

### 3.1. Demographic Data

**Figure 1:**
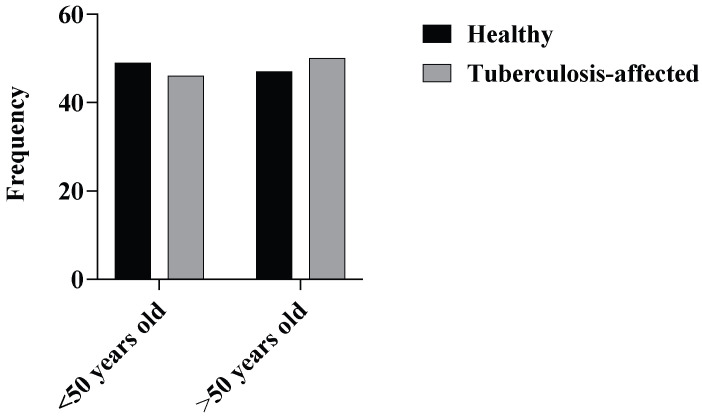
Distribution of age among the participants in the study.

A total of 192 subjects were included in this study. Data showed that the fraction of male respondents (66.14%) in the study was higher than that of the females (33.85%) (Table 1). Among 96 patients, 65 were males (67.71%) and 31 were females (32.29%), and out of 96 controls, 62 were males (64.58%) and 34 were females (35.42%). Mean age of the TB-affected respondents and unaffected respondents was found to be 56.6 years and 52.8 years, respectively (Figure 1).

Analysis of responses from the St. George’s respiratory questionnaire (SGRQ) revealed that a majority of TB-affected participants were smokers (73.95%) and reported alcohol consumption (67.70%) (Table 1). Interestingly, analysis showed that there is a statistically significant association between age and smoking status (*p* = 0.0199), with a clear trend indicating that likelihood of smoking increases with age. However, we did not find any significant association between age and status of alcohol consumption. Among the TB-affected respondents in the study, the mean body mass index (BMI) was found to be 20.62 kg/m^2^.

**Figure 2:**
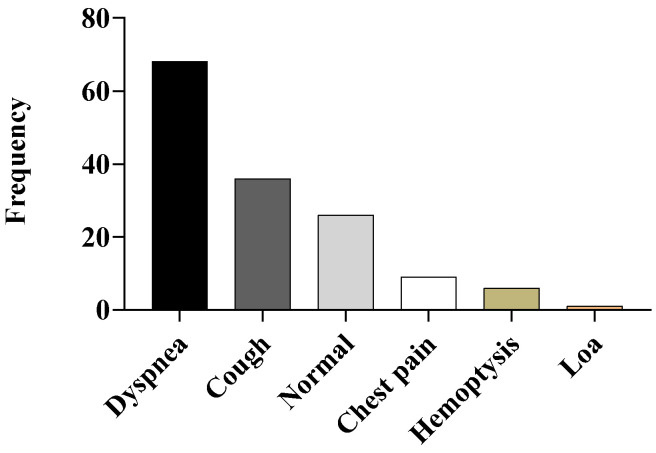
Distribution of disease symptoms among the TB-affected subjects in the study. Loa—loss of appetite.

In our study, we found that 26 (27.08%) patients had no symptoms. Among the 70 patients with symptomatic TB, 70.83% patients had dyspnea, followed by cough in 37.50%, chest pain in 9.38%, haemoptysis was found in 6.25% of the patients, and loss of appetite was seen in 1.04% of the patients (Figure 2).

Within the study subjects, 72 individuals were found to have received ATT once (75%), and 24 (25%) individuals received ATT more than once. Among the TB-affected respondents, 71 (73.96%) were found to be smokers, and 25 of them (26.04%) were non-smokers (Table 1). In comparison, the control group had 59 (61.46%) as smokers and 37 (28.54%) as 35 non-smokers.

Clinical data showed that among the 96 patients who were affected with TB, 69 (71.88%) were found to be drug sensitive and 27 (28.13%) were drug resistant. Of the 27 drug-resistant cases, incidence of rifampicin resistance was 11% (3/27), Isoniazid-resistant TB was 59% (16/27), and XDR TB was found in 30% (8/27) of the patients (Table 1).

### 3.2. Pulmonary Function Test (PFT)

**Figure 3:**
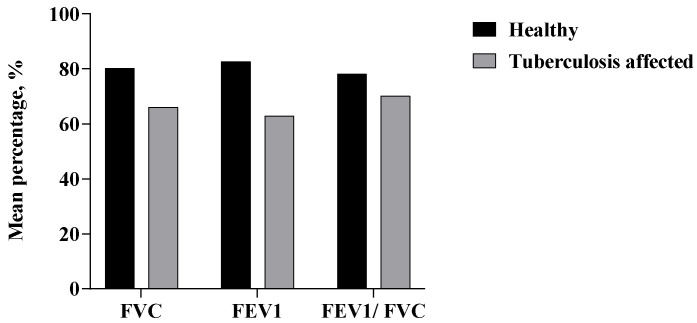
Distribution of the spirometry results (PFT values) among the healthy and TB-affected subjects in the study.

Spirometry data showed that the mean FEV1, FVC, and FEV1/FVC values in the TB patients were 62.8%, 65.97%, and 70.08%, respectively. However, among the controls in the study, the mean FEV1, FVC, and FEV1/FVC values were found to be 82.55%, 80.09%, and 78.08%, respectively (Figure 3). FEV1 was seen to be comparatively low in the TB-affected patients (62.8%) than that of the healthy subjects (82.55%). Similarly, FVC was substantially lower in the TB-affected subjects (65.97%) than that of the healthy subjects (80.09%). Similar findings were observed in the FEV1/FVC ratio among the TB-affected patients (70.08%). In summary, among the TB patients in the study, 33 (34.4%) had a mixed pattern, 28 (29.2%) had an obstructive pattern, 14 (14.5%) had restriction, and 21 (21.9%) had normal PFT.

**Figure 4:**
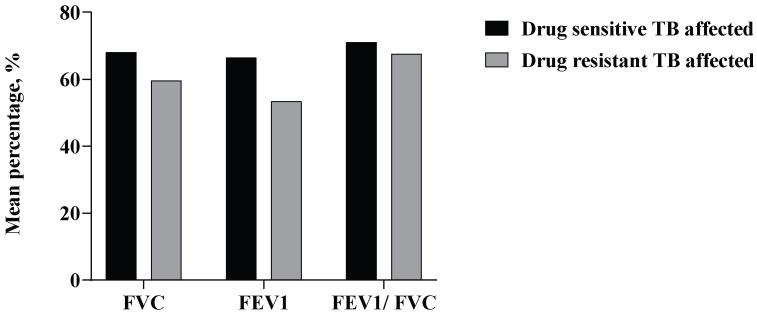
Distribution of spirometry results (PFT values) among TB-affected subjects by antimicrobial resistance status.

Among the TB-affected subjects, it was noted that those with recurrent forms of TB had relatively lower PFT values as compared to the patients with non-recurrent forms of the disease. The mean value of FVC, FEV1, and FEV1/FVC was 68.88%, 67.72%, and 72.10%, respectively, in individuals who were affected with TB once. In comparison, the mean PFT value of FVC, FEV1, and FEV1/FVC was 46.22%, 55.4%, and 63.29%, respectively, in individuals with recurrent TB. Interestingly, in the TB-affected group, cumulative means of PFT values among the DR-TB patients were comparatively lower than those of drug-sensitive TB (Figure 4).

### 3.3. St. George’s Respiratory Questionnaire (SGRQ)

**Figure 5:**
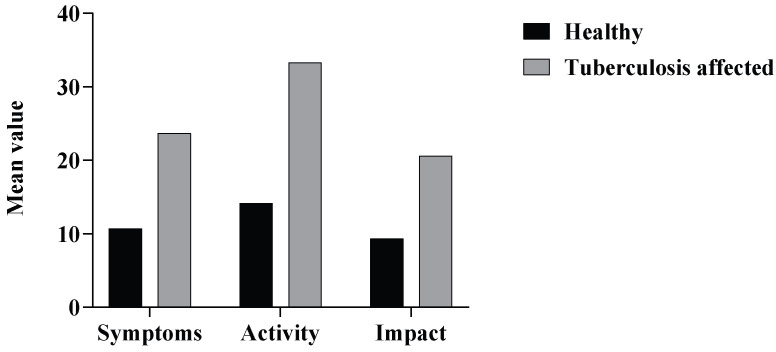
Distribution of mean SGRQ scores across the healthy and TB-affected subjects in the study.

Based on the responses from SGRQ, the mean SGRQ values for symptom, activity, and impact were 23.7, 33.32, and 20.6, respectively, in the patients who had past history of TB. In contrast, the control group in the study had mean SGRQ values of 10.71, 14.15, and 9.37, respectively, across the above three components. Statistical analysis indicated that the average score for symptoms was significantly lower (*p* = 0.032) in the healthy subjects as compared to the TB-affected respondents. Consistent with the above findings, it was seen that the average score for activity and impact was also significantly lower (*p* = 0.047 and *p* < 0.002) in the healthy subjects as compared to the TB-affected respondents (Figure 5). This indicates that the healthy subjects have a better quality of life in contrast to the TB-affected individuals in the study.

**Figure 6:**
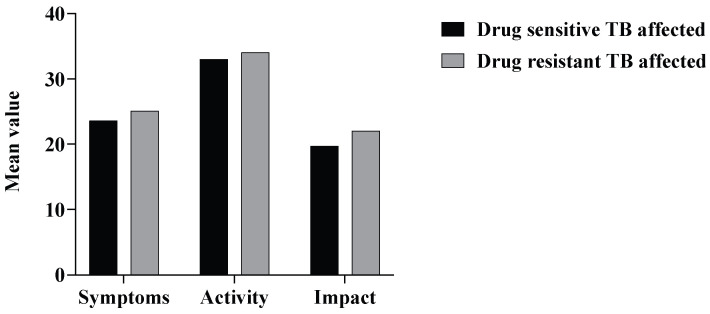
Distribution of mean SGRQ scores among TB-affected subjects by antimicrobial resistance status.

Similarly, the mean SGRQ values in the recurrent TB patients were slightly higher compared to the patients who were infected only once with TB. However, among the TB-affected subjects, the mean SGRQ values for symptoms, activity, and impact were found to have no statistically significant difference among the patients with drug-resistant and drug-sensitive forms of TB (Figure 6).

**Figure 7:**
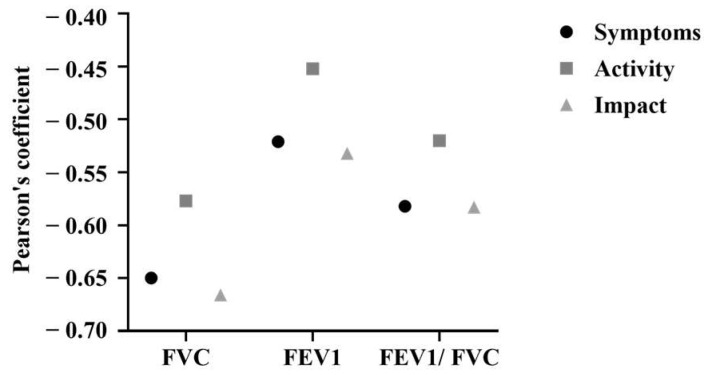
Pearson’s correlation values between PFT values and SGRQ scores among TB-affected individuals in the study.

Pearson’s correlation coefficients for mean SGRQ values and mean PFT values among the TB-affected subjects were plotted in Figure 7. In summary, all the PFT values (FEV1, FVC, and FEV1/FVC) were found to be negatively correlated with mean SGRQ values (symptoms, activity, and impact) across the subjects with a statistically significant association.

Exercise tolerance was measured using 6 MWT, as the mean distance walked by the TB patients was observed to be 327.125 m. However, healthy controls were found to have walked a mean distance of 428.02 m during the 6 MWT. Interestingly, the 6 MWT results showed that the individuals who had a history of drug-sensitive TB had better exercise tolerance (358.43 m) as compared to that of the patients who had a history of suffering from drug-resistant TB (241.11 m). The above difference could not be explained by smoking as a causative factor since the relative proportion of smokers among the DR-TB group (77.78%) is comparatively similar to that of the DS-TB group (72.46%), indicating a deficit in pulmonary function as the definitive cause of exercise tolerance. Similarly, it was observed that the patients who suffered from TB only once had a far better tolerance to exercise (355.32 m) as compared to the patients who suffered from recurrent bouts of TB, indicating poor pulmonary functional capacity.

## 4. Discussion

For many decades, tuberculosis has been known to cause physical pain, and more recently, research has increasingly focused on examining its impact on health-related quality of life (HRQoL) [39]. Quality of life (QoL) broadly encompasses the physical, emotional, and social well-being experienced by a patient during illness, reflecting their personal perspective [40]. However, these definitions vary depending on the specific condition experienced by the patient. In tuberculosis, the complexity of *Mycobacterium tuberculosis* infection results in distinct clinical states—latent infection or active disease. Additionally, TB may manifest as pulmonary or extrapulmonary, affecting different organs and thereby influencing the patient’s experience and quality of life differently [39]. Therefore, defining a single parameter to characterize HRQoL in tuberculosis is challenging due to the disease’s heterogeneity. Nonetheless, assessing quality of life indicators can provide valuable insights into the relationship between lung damage and the impact of respiratory symptoms on patients’ daily lives [41].

In our study, the mean St. George’s respiratory questionnaire (SGRQ) scores among individuals affected by tuberculosis were significantly higher compared to healthy controls, indicating a markedly poorer health-related quality of life (HRQoL) in the TB group. This finding reflects the substantial burden of respiratory symptoms and functional limitations experienced by TB patients. Our results align with previous studies that have reported elevated SGRQ scores in patients with tuberculosis and those suffering from other chronic respiratory diseases, underscoring the considerable impact of these conditions on patients’ physical, emotional, and social well-being [42,43,44,45,46].

Using reference norms for comparisons is a common approach to interpret various clinical measures, including population-based norms for children’s growth charts or healthy group norms for spirometric values [47]. Given the widespread familiarity and extensive validation of the St. George’s respiratory questionnaire (SGRQ), promoting its use among healthcare professionals could enhance the assessment of health-related quality of life (HRQoL) in respiratory patients. Many HRQoL instruments, including the SGRQ, have established normative data derived from the general population. Utilizing these population-based standards allows clinicians to interpret individual or group scores relative to a broader reference, facilitating the identification of patients with significantly impaired quality of life. This comparative approach not only aids in clinical decision-making but also supports monitoring of disease progression and evaluation of treatment efficacy over time. Significant initial impairments in lung function, exercise capacity, and quality of life can pose substantial barriers to adherence to tuberculosis treatment. This challenge is particularly pronounced during the intensive phase of therapy, when many TB control programs require patients to attend daily clinic visits for directly observed therapy (DOT). The physical limitations and symptom burden experienced by patients during this period may reduce their ability or willingness to consistently comply with treatment protocols, potentially compromising treatment outcomes and increasing the risk of disease transmission [45].

The spirometry values were represented as a percentage of the standard population values, as previously mentioned [45]. The extent of lung function impairment was categorized according to the FEV1-based standards established by the European Respiratory Society (ERS) and the American Thoracic Society (ATS) [48].

Interestingly, significant correlations were observed between PFT values and SGRQ scores among the TB-affected individuals. Similar findings were observed in various studies [42,46]. The SGRQ could, thus, serve as an additional resource for screening patients experiencing respiratory symptoms related to TB and assessing their effect on quality of life.

Spirometry serves as a conservative method to assess impairment when establishing degrees of poor health. HRQoL assessments can identify minor declines in functioning and are capable of recognizing lower degrees of poor health than those identified by spirometry [49]. The notable correlations between the PFT results and SGRQ scores found in this study could indicate the effectiveness of the HRQoL tools in evaluating health outcomes that biological measures fail to reveal.

Effective handling of disease relapse, along with the emergence of multidrug-resistant TB(MDR-TB), is associated with compliance to ATT and consequently to HRQoL [50]. A multidrug-resistant TB (MDR-TB) strain that also shows resistance to fluoroquinolones (FQs) is classified as pre-extensively drug-resistant TB (pre-XDR-TB), whereas MDR-TB strains exhibiting additional resistance to both bedaquiline (BDQ) and linezolid (LZD) are defined as extensively drug-resistant TB (XDR-TB) [51,52].

The transmission of drug-resistant TB (DR-TB) continues to be the major way through which a person becomes infected with a resistant strain [53]. Delays in treatment response and interruptions in therapy can significantly increase the risk of tuberculosis transmission within communities. Such delays not only prolong the infectious period of the patient but also contribute to the development and spread of drug-resistant TB (DR-TB) strains. Furthermore, DR-TB transmission often extends beyond household contacts, affecting broader community networks and posing substantial challenges to public health control efforts. This widespread transmission underscores the urgent need for timely diagnosis, effective treatment adherence, and robust infection control measures to limit the dissemination of resistant TB strains [54].

Managing and treating MDR-TB is a complex process; it involves high treatment expenses, the use of highly toxic anti-TB medications with possible side effects, and extended treatment duration, and it carries a higher risk of treatment failure and mortality rates [55,56,57,58]. Several studies have observed incidence of DR-TB and its impact on HRQoL among TB patients. In a study that assessed the QoL among MDR-TB cases, it was found that QoL of MDR-TB cases was comparatively worse than that of drug-sensitive TB cases [59]. Similar findings were reported in a study conducted in Yemen, where multidrug-resistant TB (MDR-TB) patients showed significant improvement in quality of life (QoL) scores upon completion of their treatment regimen. However, a sharp decline in QoL was observed during the one-year follow-up period, indicating that the benefits gained from treatment may not be sustained long-term. This pattern suggests ongoing health challenges and potential relapse or complications that continue to affect patients’ well-being after the conclusion of therapy [60]. Another study in India has also observed that QoL among MDR-TB cases was found to be poor as compared to drug-sensitive TB cases [61]. However, in our study, the mean SGRQ scores among the DR-TB patients were not statistically different from those of the drug-sensitive TB patients. This could be because varied treatment intervals in the DR-TB patients and length of sickness could influence QoL, making it difficult to differentiate the DS-TB and DR-TB patients based on health quality [60,62]. The data on HRQoL would have segregated more significantly across the DR-TB and DS-TB cases if the questionnaire survey had been conducted across different time points during or after therapy.

Given the frequent delays in diagnosis and the prolonged, often less effective nature of standard second-line treatment regimens for multidrug-resistant TB (MDR-TB) patients, there is a high likelihood of progressive lung damage occurring during the course of treatment. This ongoing pulmonary impairment can exacerbate respiratory dysfunction and negatively impact patients’ overall health outcomes, underscoring the urgent need for earlier detection and more effective therapeutic strategies to prevent further lung injury [63]. Multiple studies have evaluated pulmonary function in patients affected by multidrug-resistant TB (MDR-TB). One such study reported that 17% of MDR-TB cases exhibited airway obstruction as determined by spirometric measurements. These findings highlight the significant respiratory impairment that can persist or develop in this patient population, emphasizing the need for regular pulmonary function monitoring during and after treatment [64]. In contrast, in another study, 51% were seen to have an obstructive form among the MDR-TB cases [65]. In our study, among the DR-TB cases, only 25.92% cases were found to have an obstructive form of the disease. Interestingly, among the XDR-TB cases, 25% of the subjects were observed to have obstructive PFT values.

The rising incidence of drug-resistant tuberculosis (DR-TB) has underscored the urgent need for the development of new pharmacological agents and the repurposing of existing drugs to enhance treatment efficacy. In recent years, significant strides have been made in formulating shorter, more effective treatment regimens specifically for rifampicin-resistant TB (RR-TB), which have the potential to improve patient outcomes and reduce treatment burdens. However, the effectiveness of these novel treatment approaches is closely linked to the robustness of the healthcare system in which they are implemented. Critical features of a strong healthcare system include timely and accurate diagnostic capabilities, financial mechanisms that facilitate patient access to complete treatment courses without economic hardship, and the availability of well-trained, motivated healthcare professionals who can deliver comprehensive care and support. Furthermore, healthcare systems must provide holistic patient support, including counseling, adherence monitoring, and management of comorbid conditions, to maximize treatment success. Despite these advances, various patient-related risk factors continue to impede optimal outcomes. HIV co-infection, for example, complicates treatment and increases morbidity; alcohol consumption can interfere with medication adherence and liver function; and overall challenges in maintaining consistent treatment adherence remain significant barriers. Addressing both systemic healthcare challenges and individual patient risk factors is, therefore, essential to improve the prognosis and control of DR-TB globally [66].

It is important to note that the TB patient group and the healthy controls in the study were not matched with their data on geographical region, economic status, comorbidities, and environmental factors. This could have introduced potential confounding in the differences observed between the healthy and TB patient groups in QoL scores and pulmonary function values. Another major limitation in this study was not utilizing chest radiography to assess the anatomical and pathological changes in the TB-affected respondents. This would have been especially valuable to compare any structural defects in the patients with DR-TB cases with those of the drug-resistant TB cases and their relationship with pulmonary function and QoL.

## 5. Conclusions

The study shows the significant long-term impact of TB on pulmonary function, exercise capacity, and health-related quality of life (HRQoL), particularly among patients with drug-resistant and recurrent TB. Despite successful microbiological treatment, many patients continue to experience considerable pulmonary impairment, as evidenced by reduced spirometry values, lower six-minute walk test distances, and elevated St. George’s respiratory questionnaire (SGRQ) scores. These impairments are more pronounced in those with drug-resistant TB and recurrent infections, emphasizing the need for focused post-treatment monitoring in these populations.

Our findings suggest that routine incorporation of pulmonary function tests, HRQoL evaluations, and exercise tolerance assessments in post-TB care could facilitate early identification of patients at risk of chronic respiratory disability. Furthermore, targeted interventions, including pulmonary rehabilitation, smoking cessation programs, and psychosocial support, may be essential to improve both functional outcomes and overall quality of life. Future research should explore tailored therapeutic strategies to mitigate long-term sequelae and optimize recovery in TB survivors. Addressing these aspects comprehensively will be critical in reducing the global burden of TB-related morbidity and improving patient-centered care.

## Figures and Tables

**Table 1:** Characteristics of overall TB-affected subjects (n = 96) in the study.

Parameters	Number of Patients (%)
Age	
I. 35–45 years	9 (9.37%)
II. 45–55 years	46 (47.92%)
III. 55–65 years	22 (22.92%)
IV. 65–75 years	19 (19.79%)
Total Male respondents	65 (67.71%)
Total Female respondents	31 (32.29%)
Smoking habit	71 (73.95%)
I. Males	48 (67.60%)
II. Females	23 (32.40%)
Alcohol consumption	65 (67.71%)
I. Males	43 (66.15%)
II. Females	22 (33.85%)
Recurrent TB cases	22 (22.91%)
Incidence of DR-TB	27 (28.12%)
(within 27 DR-TB-affected respondents)	
I. Isoniazid resistance	16/27 (59.26%)
II. Rifampicin resistance	3/27 (11.11%)
III. XDR-TB	8/27 (29.63%)

TB—tuberculosis; DR-TB—drug-resistant tuberculosis; XDR-TB—extensively drug-resistant tuberculosis.

## Data Availability

The original contributions presented in this study are included in the article. Further inquiries can be directed to the corresponding authors.

## References

[1] World Health Organization (WHO) Tuberculosis. https://www.who.int/news-room/fact-sheets/detail/tuberculosis.

[2] Banerjee S., Bandyopadhyay K., Taraphdar P., Dasgupta A. (2019). Effect of DOTS on Quality of Life among Tuberculosis Patients: A Follow-up Study in a Health District of Kolkata. J. Fam. Med. Prim. Care.

[3] Bauer M., Ahmed S., Benedetti A., Greenaway C., Lalli M., Leavens A., Menzies D., Vadeboncoeur C., Vissandjée B., Wynne A. (2015). Health-Related Quality of Life and Tuberculosis: A Longitudinal Cohort Study. Health Qual. Life Outcomes.

[4] The End TB Strategy. https://www.who.int/teams/global-programme-on-tuberculosis-and-lung-health/the-end-tb-strategy.

[5] India TB Report 2022—Central Tuberculosis Division. https://tbcindia.mohfw.gov.in/2023/06/06/india-tb-report-2022/.

[6] Rajpal S., Arora V.K. (2020). Latent TB (LTBI) Treatment: Challenges in India with an Eye on 2025. Indian J. Tuberc..

[7] Sharma N., Basu S., Chopra K.K. (2019). Achieving TB Elimination in India: The Role of Latent TB Management. Indian J. Tuberc..

[8] Chauhan A., Parmar M., Dash G.C., Solanki H., Chauhan S., Sharma J., Sahoo K.C., Mahapatra P., Rao R., Kumar R. (2023). The Prevalence of Tuberculosis Infection in India: A Systematic Review and Meta-Analysis. Indian J. Med. Res..

[9] Mazurek G.H., Jereb J., Lobue P., Iademarco M.F., Metchock B., Vernon A., Division of Tuberculosis Elimination, National Center for HIV, STD, and TB Prevention, Centers for Disease Control and Prevention (CDC) (2005). Guidelines for Using the QuantiFERON-TB Gold Test for Detecting Mycobacterium Tuberculosis Infection, United States. Morb. Mortal. Wkly. Rep. Recomm. Rep..

[10] World Health Organization (WHO) Drug-resistant TB. https://www.who.int/teams/global-programme-on-tuberculosis-and-lung-health/tb-reports/global-tuberculosis-report-2022/tb-disease-burden/2-3-drug-resistant-tb.

[11] Seung K.J., Keshavjee S., Rich M.L. (2015). Multidrug-Resistant Tuberculosis and Extensively Drug-Resistant Tuberculosis. Cold Spring Harb. Perspect. Med..

[12] Chushkin M.I., Ots O.N. (2017). Impaired Pulmonary Function after Treatment for Tuberculosis: The End of the Disease?. J. Bras. Pneumol..

[13] Viana Mancuzo E., Martins Netto E., Sulmonett N., de Souza Viana V., Croda J., Lineu Kritski A., Carvalho de Queiroz Mello F., de Souza Elias Nihues S., Rosas Sodre Azevedo K., Spíndola de Miranda S. (2020). Spirometry Results after Treatment for Pulmonary Tuberculosis: Comparison between Patients with and without Previous Lung Disease: A Multicenter Study. J. Bras. Pneumol..

[14] Olufemi A.O., Chikaodinaka A.A., Abimbola P., Oluwatoyin A.T., Oluwafunmilola A., Fasanmi K.T., Efosa E.G. (2018). Health-Related Quality of Life (HRQoL) Scores Vary with Treatment and May Identify Potential Defaulters during Treatment of Tuberculosis. Malawi Med. J..

[15] Yasobant S., Nazli Khatib M., Syed Z.Q., Gaidhane A.M., Shah H., Narkhede K., Bhavsar P., Patel J., Sinha A., Puwar T. (2022). Health-Related Quality of Life (HRQoL) of Patients with Tuberculosis: A Review. Infect. Dis. Rep..

[16] Duong M., Usman A., Ma J., Xie Y., Huang J., Zaman M., Dragoman A., Jiatong Chen S., Farooqi M., Raina P. (2022). Associations between Lung Function and Physical and Cognitive Health in the Canadian Longitudinal Study on Aging (CLSA): A Cross-Sectional Study from a Multicenter National Cohort. PLoS Med..

[17] Smirnova N., Lowers J., Magee M.J., Auld S.C., Hunt W.R., Fitzpatrick A., Lama V., Kavalieratos D. (2023). Pulmonary Function and Quality of Life in Adults with Cystic Fibrosis. Lung.

[18] Ribeiro Moço V.J., Lopes A.J., dos Santos Vigário P., de Almeida V.P., de Menezes S.L.S., Guimarães F.S. (2015). Pulmonary Function, Functional Capacity and Quality of Life in Adults with Cystic Fibrosis. Rev. Port. Pneumol..

[19] Vanaparthy R., Mota P., Khan R., Ehsan M., Qureshi A., ZuWallack R., Leidy N. (2015). A Longitudinal Assessment of Sleep Variables during Exacerbations of Chronic Obstructive Pulmonary Disease. Chron. Respir. Dis..

[20] Baghai-Ravary R., Quint J.K., Goldring J.J.P., Hurst J.R., Donaldson G.C., Wedzicha J.A. (2009). Determinants and Impact of Fatigue in Patients with Chronic Obstructive Pulmonary Disease. Respir. Med..

[21] Torres-Sánchez I., Cabrera-Martos I., Díaz-Pelegrina A., Valenza-Demet G., Moreno-Ramírez M.P., Valenza M.C. (2017). Physical and Functional Impairment During and After Hospitalization in Subjects with Severe COPD Exacerbation. Respir. Care.

[22] Oliveira C.C., Lee A.L., McGinley J., Anderson G.P., Clark R.A., Thompson M., Clarke S., Baker T., Irving L.B., Denehy L. (2017). Balance and Falls in Acute Exacerbation of Chronic Obstructive Pulmonary Disease: A Prospective Study. COPD J. Chronic Obstr. Pulm. Dis..

[23] Kania A., Krenke R., Kuziemski K., Czajkowska-Malinowska M., Celejewska-Wójcik N., Kuznar-Kaminska B., Farnik M., Bokiej J., Miszczuk M., Damps-Konstanska I. (2018). Distribution and Characteristics of COPD Phenotypes & ndash; Results from the Polish Sub-Cohort of the POPE Study. Int. J. Chron. Obstruct. Pulmon. Dis..

[24] Teixeira P.J.Z., Porto L., Kristensen C.H., Santos A.H., Menna-Barreto S.S., Prado-Lima P.A.S. (2015). Do Post-Traumatic Stress Symptoms and Exacerbations in COPD Patients. COPD J. Chronic Obstr. Pulm. Dis..

[25] Hansel N.N., Wu A.W., Chang B., Diette G.B. (2004). Quality of Life in Tuberculosis: Patient and Provider Perspectives. Qual. Life Res..

[26] Kelly P. (1999). Isolation and Stigma: The Experience of Patients with Active Tuberculosis. J. Community Health Nurs..

[27] Aggarwal A.N. (2019). Quality of Life with Tuberculosis. J. Clin. Tuberc. Other Mycobact. Dis..

[28] Cremers A.L., de Laat M.M., Kapata N., Gerrets R., Klipstein-Grobusch K., Grobusch M.P. (2015). Assessing the Consequences of Stigma for Tuberculosis Patients in Urban Zambia. PLoS ONE.

[29] Craig G.M., Daftary A., Engel N., O’Driscoll S., Ioannaki A. (2017). Tuberculosis Stigma as a Social Determinant of Health: A Systematic Mapping Review of Research in Low Incidence Countries. Int. J. Infect. Dis..

[30] Orhan Aydin I., Uluşahin A. (2001). Depression, Anxiety Comorbidity, and Disability in Tuberculosis and Chronic Obstructive Pulmonary Disease Patients: Applicability of GHQ-12. Gen. Hosp. Psychiatry.

[31] Bauer M., Leavens A., Schwartzman K.A. (2013). Systematic Review and Meta-Analysis of the Impact of Tuberculosis on Health-Related Quality of Life. Qual. Life Res..

[32] Rajeswari R., Muniyandi M., Balasubramanian R., Narayanan P.R. (2005). Perceptions of Tuberculosis Patients about Their Physical, Mental and Social Well-Being: A Field Report from South India. Soc. Sci. Med..

[33] Dar S.A., Shah N.N., Wani Z.A., Nazir D. (2019). A Prospective Study on Quality of Life in Patients with Pulmonary Tuberculosis at a Tertiary Care Hospital in Kashmir, Northern India. Indian J. Tuberc..

[34] Kisaka S.M.B., Rutebemberwa E., Kasasa S., Ocen F., Nankya-Mutyoba J. (2016). Does Health-Related Quality of Life among Adults with Pulmonary Tuberculosis Improve across the Treatment Period? A Hospital-Based Cross Sectional Study in Mbale Region, Eastern Uganda. BMC Res. Notes.

[35] Meghji J., Auld S.C., Bisson G.P., Khosa C., Masekela R., Navuluri N., Rachow A. (2025). Post-Tuberculosis Lung Disease: Towards Prevention, Diagnosis, and Care. Lancet Respir. Med..

[36] Ferrer M., Villasante C., Alonso J., Sobradillo V., Gabriel R., Vilagut G., Masa J.F., Viejo J.L., Jiménez-Ruiz C.A., Miravitlles M. (2002). Interpretation of Quality of Life Scores from the St George’s Respiratory Questionnaire. Eur. Respir. J..

[37] Jones P.W. (2005). St. George’s Respiratory Questionnaire: MCID. COPD J. Chronic Obstr. Pulm. Dis..

[38] Daniels K.J., Irusen E., Pharaoh H., Hanekom S. (2019). Post-Tuberculosis Health-Related Quality of Life, Lung Function and Exercise Capacity in a Cured Pulmonary Tuberculosis Population in the Breede Valley District, South Africa. S. Afr. J. Physiother..

[39] Peddireddy V. (2016). Quality of Life, Psychological Interventions and Treatment Outcome in Tuberculosis Patients: The Indian Scenario. Front. Psychol..

[40] Roila F., Cortesi E. (2001). Quality of Life as a Primary End Point in Oncology. Ann. Oncol..

[41] Carreto-Binaghi L.E., Sartillo-Mendoza L.G., Muñoz-Torrico M., Guzmán-Beltrán S., Carranza C., Torres M., González Y., Juárez E. (2023). Serum Pro-Inflammatory Biomarkers Associated with Improvement in Quality of Life in Pulmonary Tuberculosis. Front. Immunol..

[42] Pasipanodya J.G., Miller T.L., Vecino M., Munguia G., Bae S., Drewyer G., Weis S.E. (2007). Using the St. George Respiratory Questionnaire to Ascertain Health Quality in Persons with Treated Pulmonary Tuberculosis. Chest.

[43] Jones P.W., Bosh T.K. (1997). Quality of Life Changes in COPD Patients Treated with Salmeterol. Am. J. Respir. Crit. Care Med..

[44] Kastien-Hilka T., Rosenkranz B., Sinanovic E., Bennett B., Schwenkglenks M. (2017). Health-Related Quality of Life in South African Patients with Pulmonary Tuberculosis. PLoS ONE.

[45] Maguire G.P., Anstey N.M., Ardian M., Waramori G., Tjitra E., Kenangalem E., Handojo T., Kelly P.M. (2009). Pulmonary Tuberculosis, Impaired Lung Function, Disability and Quality of Life in a High-Burden Setting. Int. J. Tuberc. Lung Dis..

[46] Vashakidze S.A., Kempker J.A., Jakobia N.A., Gogishvili S.G., Nikolaishvili K.A., Goginashvili L.M., Magee M.J., Kempker R.R. (2019). Pulmonary Function and Respiratory Health after Successful Treatment of Drug-Resistant Tuberculosis. Int. J. Infect. Dis..

[47] Meguro M., Barley E.A., Spencer S., Jones P.W. (2007). Development and Validation of an Improved, COPD-Specific Version of the St. George Respiratory Questionnaire. Chest.

[48] Pellegrino R., Viegi G., Brusasco V., Crapo R.O., Burgos F., Casaburi R., Coates A., van der Grinten C.P.M., Gustafsson P., Hankinson J. (2005). Interpretative Strategies for Lung Function Tests. Eur. Respir. J..

[49] Curtis J.R., Patrick D.L. (2003). The Assessment of Health Status among Patients with COPD. Eur. Respir. J..

[50] Munro S.A., Lewin S.A., Smith H.J., Engel M.E., Fretheim A., Volmink J. (2007). Patient Adherence to Tuberculosis Treatment: A Systematic Review of Qualitative Research. PLoS Med..

[51] Clinical Overview of Drug-Resistant Tuberculosis Disease. https://www.cdc.gov/tb/hcp/clinical-overview/drug-resistant-tuberculosis-disease.html.

[52] Dookie N., Ngema S.L., Perumal R., Naicker N., Padayatchi N., Naidoo K. (2022). The Changing Paradigm of Drug-Resistant Tuberculosis Treatment: Successes, Pitfalls, and Future Perspectives. Clin. Microbiol. Rev..

[53] Faustini A. (2006). Risk Factors for Multidrug Resistant Tuberculosis in Europe: A Systematic Review. Thorax.

[54] Grandjean L., Crossa A., Gilman R.H., Herrera C., Bonilla C., Jave O., Cabrera J.L., Martin L., Escombe A.R., Moore D.A.J. (2011). Tuberculosis in Household Contacts of Multidrug-Resistant Tuberculosis Patients. Int. J. Tuberc. Lung Dis..

[55] Chung-Delgado K., Guillen-Bravo S., Revilla-Montag A., Bernabe-Ortiz A. (2015). Mortality among MDR-TB Cases: Comparison with Drug-Susceptible Tuberculosis and Associated Factors. PLoS ONE.

[56] Bonilla C.A., Crossa A., Jave H.O., Mitnick C.D., Jamanca R.B., Herrera C., Asencios L., Mendoza A., Bayona J., Zignol M. (2008). Management of Extensively Drug-Resistant Tuberculosis in Peru: Cure Is Possible. PLoS ONE.

[57] Santha T., Garg R., Frieden T.R., Chandrasekaran V., Subramani R., Gopi P.G., Selvakumar N., Ganapathy S., Charles N., Rajamma J. (2002). Risk Factors Associated with Default, Failure and Death among Tuberculosis Patients Treated in a DOTS Programme in Tiruvallur District, South India, 2000. Int. J. Tuberc. Lung Dis..

[58] Suárez P.G., Floyd K., Portocarrero J., Alarcón E., Rapiti E., Ramos G., Bonilla C., Sabogal I., Aranda I., Dye C. (2002). Feasibility and Cost-Effectiveness of Standardised Second-Line Drug Treatment for Chronic Tuberculosis Patients: A National Cohort Study in Peru. Lancet.

[59] Sharma R., Yadav R., Sharma M., Saini V., Koushal V. (2014). Quality of Life of Multi Drug Resistant Tuberculosis Patients: A Study of North India. Acta Med. Iran..

[60] Jaber A.A.S., Ibrahim B. (2019). Health-Related Quality of Life of Patients with Multidrug-Resistant Tuberculosis in Yemen: Prospective Study. Health Qual. Life Outcomes.

[61] Venkatesh U., Sharma A., Srivastava D.K., Durga R. (2022). Health-Related Quality of Life of Multidrug-Resistant Tuberculosis Patients: A Study of Eastern Uttar Pradesh, India. Indian J. Tuberc..

[62] Gare K.P., Sebakeng M., Molefi M. (2024). The Health-Related Quality of Life of Drug-Resistant Tuberculosis Patients Receiving Treatment in Botswana. BMC Infect. Dis..

[63] Dheda K., Gumbo T., Maartens G., Dooley K.E., McNerney R., Murray M., Furin J., Nardell E.A., London L., Lessem E. (2017). The Epidemiology, Pathogenesis, Transmission, Diagnosis, and Management of Multidrug-Resistant, Extensively Drug-Resistant, and Incurable Tuberculosis. Lancet Respir. Med..

[64] de Vallière S., Barker R.D. (2004). Residual Lung Damage after Completion of Treatment for Multidrug-Resistant Tuberculosis. Int. J. Tuberc. Lung Dis..

[65] Byrne A.L., Marais B.J., Mitnick C.D., Garden F.L., Lecca L., Contreras C., Yauri Y., Garcia F., Marks G.B. (2017). Chronic Airflow Obstruction after Successful Treatment of Multidrug-Resistant Tuberculosis. ERJ Open Res..

[66] Kurbatova E.V., Taylor A., Gammino V.M., Bayona J., Becerra M., Danilovitz M., Falzon D., Gelmanova I., Keshavjee S., Leimane V. (2012). Predictors of Poor Outcomes among Patients Treated for Multidrug-Resistant Tuberculosis at DOTS-plus Projects. Tuberculosis.

